# A palatable hyperlipidic diet causes obesity and affects brain glucose metabolism in rats

**DOI:** 10.1186/1476-511X-10-168

**Published:** 2011-09-23

**Authors:** Debora Estadella, Lila M Oyama, Allain A Bueno, Carlos A Habitante, Gabriel I Souza, Eliane B Ribeiro, Caio SM Motoyama, Claudia M Oller do Nascimento

**Affiliations:** 1 Disciplina de Fisiologia da Nutrição, Departamento de Fisiologia, Universidade Federal de São Paulo, São Paulo, SP, Brazil; 2 Institute of Brain Chemistry and Human Nutrition, London Metropolitan University, London, UK; 3 Instituto de Ciências Biológicas e da Saúde, Campus Universitário do Araguaia, Universidade Federal do Mato Grosso, MT, Brazil

## Abstract

**Background:**

We have previously shown that either the continuous intake of a palatable hyperlipidic diet (H) or the alternation of chow (C) and an H diet (CH regimen) induced obesity in rats. Here, we investigated whether the time of the start and duration of these feeding regimens are relevant and whether they affect brain glucose metabolism.

**Methods:**

Male Wistar rats received C, H, or CH diets during various periods of their life spans: days 30-60, days 30-90, or days 60-90. Experiments were performed the 60^th ^or the 90^th ^day of life. Rats were killed by decapitation. The glucose, insulin, leptin plasma concentration, and lipid content of the carcasses were determined. The brain was sliced and incubated with or without insulin for the analysis of glucose uptake, oxidation, and the conversion of [1-^14^C]-glucose to lipids.

**Results:**

The relative carcass lipid content increased in all of the H and CH groups, and the H30-60 and H30-90 groups had the highest levels. Groups H30-60, H30-90, CH30-60, and CH30-90 exhibited a higher serum glucose level. Serum leptin increased in all H groups and in the CH60-90 and CH30-90 groups. Serum insulin was elevated in the H30-60, H60-90, CH60-90, CH30-90 groups. Basal brain glucose consumption and hypothalamic insulin receptor density were lower only in the CH30-60 group. The rate of brain lipogenesis was increased in the H30-90 and CH30-90 groups.

**Conclusion:**

These findings indicate that both H and CH diet regimens increased body adiposity independent treatment and the age at which treatment was started, whereas these diets caused hyperglycemia and affected brain metabolism when started at an early age.

## Introduction

Glucose is considered to be the major nutrient for cells of the adult nervous system. A small portion of glucose is used for biosynthesis pathways. Dhopeshwarkar and Subramanian [1] showed that intracranially administered D- [U-^14^C] glucose was incorporated in saturated fatty acids and, to a lesser extent, in monounsaturated fatty acids. Poor glucose metabolism has also been related to memory problems [2].

In a previous review, it was reported that high-fat diets, especially diets enriched with saturated fatty acids, impaired the learning and memory of rodents and that these effects could be related to insulin resistance and impaired glucose metabolism [3,4]. However, polyunsaturated fatty acids improve cognitive function and are beneficial for the prevention of cognitive decline [5]. Kaiyala et al. [6] reported a reduction of insulin uptake and clearance by the central nervous system in dogs that were fed a hyperlipidic diet.

Insulin receptors are expressed in several regions of the central nervous system [7,8]. Experimental evidence has demonstrated the important role of the insulin central receptors as normal energy balance controllers [9-11] in addition to their role in neuronal growth and differentiation during development [12,13]. According to Carvalheira et al. [14], insulin modulates the leptin signal transduction pathway in the hypothalamus, playing a synergistic role in regulating food intake and controlling weight.

Previous experiments have shown that rats fed a high-fat diet had an increased fat mass percentage even when the energy intake was not elevated [15,16]. Likewise, we previously demonstrated that the continuous intake of a hyperlipidic palatable diet and the alternation of a high-fat intake with periods of chow intake for 8 weeks caused obesity and affected the lipid metabolism of rats in a similar way, although the energy intake remained unchanged (KJ) [17].

In these experimental models, obesity was accompanied by hyperleptinemia, normoglycemia, hyperinsulinemia and insulin resistance. The age, gender weight, and period on high-fat diets of the animals influenced their metabolism [16,18-20].

There are controversial results regarding the effect of hyperlipidic diets on the expression of leptin receptors (Ob-Rb and Ob-Ra) in the central nervous system. Madiehe et al. [21] suggested that a decrease in the levels of both the long and short form of the hypothalamus leptin receptors could induce leptin resistance in hyperlipidic-fed rats. In mice given a high-fat diet, Lin et al. [22] reported an increase in mRNA leptin receptor expression after 8 weeks and a reduction of this expression after 19 weeks. Boado et al. [23] detected OB-Ra up-regulation in the blood-brain-barrier of the rats after 14 weeks of high-fat feeding.

The suckling-weaning transition of rats is accompanied by marked dietary changes. Suckling rats eat a very high-fat, low-carbohydrate diet, and this is replaced by a low-fat, high-carbohydrate diet at the time of weaning [24]. The utilization of substrates by the brain depends on several factors: 1) the plasma concentrations of the substrate, 2) the permeability of the blood-brain barrier to the substrates, and 3) the intrinsic capability of the brain to utilize the substrates [25].

The present study was designed to investigate whether the continuous feeding of a palatable hyperlipidic diet or cycling a hyperlipidic diet with a chow diet induced obesity. We evaluated whether the time of the start and duration of these feeding regimens were relevant and whether they affected brain glucose metabolism.

## Materials and Methods

### Animals

The Experimental Research Committee of the Federal University of São Paulo approved the procedures used in the experiments presented here (CEP n°0446/03).

Male Wistar rats supplied by the animal care facility of the Physiology Department of the Federal University of São Paulo were assigned to 8 groups according to diet composition: (C) receiving a chow diet, (H) receiving a palatable hyperlipidic diet and (CH) receiving a cycled diet (cycles alternating weekly between a chow diet and a palatable hyperlipidic diet). The time of the start and duration of these feeding regimens were also studied using the following groups: diet received from the 30^th ^to 60^th ^day of life (30-60), diet received from the 30^th ^to 90^th ^day of life (30-90) and diet received from the 60^th ^to 90^th ^day of life (60-90).

All groups were maintained in a room at 23°C ± 1°C with light from 7:00 AM to 7:00 PM and received food and water *ad libitum*. Body weight and food intake were measured weekly. Spillage was minimized due to the use of specially designed covered food cups. All the animals were sacrificed in the fed state in the early morning to avoid chronobiological variations. The animals were acclimatized in a quiet room next to the laboratory under subdued light for 1 h and were taken to the laboratory individually to minimize stress.

### Preparation of the palatable hyperlipidic diet

The palatable hyperlipidic diet consists of commercial rat chow plus peanuts, milk chocolate, and sweet biscuits in the proportion of 3:2:2:1. All components were powdered and mixed. This diet was composed of 20% protein, 20% fat, and 40% carbohydrate. The control diet contained 20% protein, 4.5% fat, and 55% carbohydrate. The caloric densities of the diets were determined using an IKA-C400 adiabatic calorimeter. The caloric density was 21.40 kJ/g (35% of calories from fat) for the palatable hyperlipidic diet and 17.03 kJ/g for the chow diet.

### Fatty acid composition of the diets

Five hundred milligrams of the diets were treated with 2.0 mL of methanol:benzene (4:1, v/v) followed by 200 μL of acetyl chloride under light agitation [26]. Fatty acid methyl esters were separated (SP2560 column, Supelco, Bellefonte, PA, USA) and quantified using gas-liquid chromatography with an ionizable flame detector (Perkin Elmer, Wellesley, MA, USA) and hydrogen as the carrier gas. The injection and detection temperatures were 260°C and 280°C, respectively. The run temperature started at 135°C and increased to 195°C during a run time of 45 min. The fatty acid composition of the diets is presented in Table 1.

**Table 1:** Fatty acid profile of the diets, as percent of total lipid content

	Total Fatty Acid (%)	
Fatty Acid	C	H
**C14:0**	0.11	0.53
**C16:0**	13.37	17.85
**C16:1n7**	0.21	0.15
**C17:0**	0.09	0.12
**C18:0**	2.95	11.05
**C18:1n9**	24.13	36.59
**C18:2n6**	49.45	27.27
**C18:3n3**	3.73	0.49
**C20:0**	0.42	1.19
**C22:0**	0.04	2.14
**C22:2n6**	0.04	0.04
**C23:0**	0.07	0.04
**C22:5n3**	0.22	0.14
**C24:0**	0.34	1.15

**SFA**	17.4	34.06
**MUFA**	25.59	38.00
**PUFA**	56.97	27.95

Control diet (C) and palatable hyperlipidic diet (H)

SFA. saturated fatty acids; MUFA. monounsaturated fatty acids; PUFA. polyunsaturated fatty acids. * Data are means ± SEMs of three determinations.

### Experimental Procedure

Experiments were performed on the 60^th ^or 90^th ^day of the rats' lives, and the rats were killed by decapitation. Trunk blood was collected, serum was obtained via centrifugation, and aliquots were taken to measure the concentration of glucose using commercials kits from Labtest Diagnostic S.A (Minas Gerais, Brazil) and the concentrations of insulin and leptin using ELISA (Linco Research, Inc., USA).

The brain was removed, the cerebellum and brain stem were discarded, and the remaining brain was sliced as described by Liu et al. (2005) [27] and immediately incubated to determine the brain glucose consumption, glucose oxidation, and lipid synthesis. Another set of rats was used for the Western blotting analysis. The carcasses were weighed and stored in a freezer (-20°C) for lipid content analysis.

### Carcass lipid content determination

The carcasses were eviscerated, weighed, and stored at -20°C. The lipid content was measured as described by Stansbie et al. [28] and standardized using the method described by Oller do Nascimento and Williamson [29]. Briefly, the eviscerated carcass was autoclaved at 120°C for 90 min and then homogenized with double its mass of water. Triplicate aliquots of this homogenate were weighed and digested in 3 ml of 30% KOH and 3 ml of ethanol for at least 2 h at 70°C in capped tubes. After cooling, 2 ml of 12 N H2SO4 were added, and the sample was washed three times with petroleum ether for lipid extraction. The results are expressed as grams of lipid/100 g of carcass.

### Brain incubation

The total brain was sliced and used for the incubation procedure (~100 mg). Incubations were performed at 37°C in 25 ml Erlenmeyer flasks equipped with a central well. The incubation medium consisted of 2.0 ml of Krebs-Henseleit buffer containing 5 mM glucose plus 0.04 μCi ^14^C-glucose with or without 0.5 U/ml insulin, as it has been previously demonstrated that chronic hyperinsulinemia could alter the glucose utilization of several brain regions [30]. The flasks were continuously shaken and flushed with carbogenium (O_2_/CO_2_, 95/5%) during the incubation period.

After 1 hour, incubation was stopped by the addition of 0.5 ml of 4 N H_2_SO_4 _to the main well, and 0.3 ml of NaOH (1 N) was then added to the central well for ^14^CO_2 _collection. After two hours, the brain slices were removed, and the total lipids were extracted with 10 ml of chloroform:methanol (2:1). The radioactivity of this extract represented the conversion of [1-^14^C]-glucose to lipids. The rates of conversion of [1-^14^C]-glucose to ^14^CO_2 _and incorporation into the lipid fraction were expressed as μmol/h × g of tissue. The incubation medium was used for the determination of the brain glucose consumption using commercials kits from Labtest Diagnostic S.A. These methods have been previously described [31].

### Western blot analysis

The rat cranium was opened, and the hypothalamus was quickly removed, minced coarsely and immediately homogenized in 1 mL of buffer [100 mM Tris (pH 7.6), 10 mM Na_3_VO_4_, 2 mM PMSF, 10 mM EDTA, 100 mM NaF, 10 mM Na_4_P_2_O_7_, 0.1 mg/mL × aprotinin] using an ULTRA TURAX IKA T-18 BASIC generator operated at maximum speed for 30 s. Then, 10% Triton X-100 was added, and the solution was clarified by centrifugation (12.000 rpm, 20 min, at 4°C).

The total protein content from the entire tissue extraction was determined using standard BSA, following previously described methods [32]. One hundred micrograms of hypothalamus total protein was loaded onto a sodium dodecyl sulfate-polyacrilamide gel (5% stacking gel; 10% running gel), separated by electrophoresis and then electroblotted onto nitrocellulose membranes (Hybond-C Extra, Amersham) using a wet electroblotter (Bio-Rad, CA, USA). After blotting, the membranes were blocked in Tris-buffered saline (TBS)-Tween buffer, pH 7.5 (20 mM Tris/500 mM NaCl/0.05% Tween-20) containing 10% skimmed milk powder for 2 h and then exposed to either anti-insulin receptor (IR) or anti-leptin receptor (OB-R) at a dilution of 1/200 in TBS-Tween buffer (pH 7.5) containing 1% BSA for 2 h. The membranes then were washed and incubated with anti-rabbit Ig or anti-mouse Ig conjugated to horseradish peroxidase and diluted to 1/1000 in the same buffer for 1 h. After a series of washes in TBS-Tween buffer, the protein bands were visualized by chemiluminescence with an ECL luminescence kit (Amersham) and exposure to Hyper-film ECL (Amersham). The size of the protein bands was determined using electrophoresis color markers. The antibodies against IR (sc-711) and OB-R (sc-8391) were obtained from Santa Cruz Biotechnology (Santa Cruz, Calif., USA), and the anti-rabbit Ig and anti-mouse Ig conjugated to horseradish peroxidase were obtained from Sigma (USA). A quantitative analysis of the blots was performed using Image J software.

### Statistical Analysis

The results are expressed as the mean ± standard error of means. Statistical comparisons were carried out using analysis of variance (one-way ANOVA) following by a post-hoc analysis (Tukey test) to compare the effects of the different diets among animals of the same age. The level of significance was set at p < 0.05.

## Results

### Body Weight, Energy Intake and Carcass lipid content

The increment in body weight was not significantly different among the C30-60, H30-60 and CH30-60 groups. When the animals were fed a palatable hyperlipidic diet or the cycled diet from the 30^th ^to 90^th ^or from the 60^th ^to 90^th ^day of life, we observed a significant increase in body weight compared to the respective C groups (Table 2).

**Table 2:** Increment in body weight (g), total caloric intake (kJ/100 g b.w gain) and carcass relative lipid content (g/100 g)

		C	H	CH
**Increment in body weight **(g)	**30-60**	118.1 ± 4.8^a^	124.7 ± 8.2ª	126.8 ± 6.8ª
	**60-90**	77.1 ± 3.1^a^	91.9 ± 3.2^b^	103.9 ± 3.3^c^
	**30-90**	198.1 ± 9.9^a^	207.8 ± 7.4^ab^	237.2 ± 13.5^b^

**Total caloric intake** (kJ/100 g b.w)	**30-60**	7017.9 ± 656.0^a^	6882.7 ± 464.7ª	6875.1 ± 357.0ª
	**60-90**	15282.7 ± 656.0^a^	12662.3 ± 408.1^b^	11057.6 ± 359.7^b^
	**30-90**	10289.9 ± 642.9^a^	9675.2 ± 401.6^ab^	8162.0 ± 369.9^b^

**Carcass Relative lipid content**(g/100 g)	**30-60**	2.32 ± 0.18^a^	5.48 ± 0.35^b^	3.94 ± 0.29^c^
	**60-90**	2.65 ± 0.17^a^	4.26 ± 0.34^b^	3.75 ± 0.32^b^
	**30-90**	2.65 ± 0.17^a^	3.94 ± 0.26^b^	3.49 ± 0.21^b^

Control rats (C), rats treated with palatable hyperlipidic diet (H) and rats treated with food cycles alternating chow and palatable hyperlipidic diets (CH). The animals received these diets from 30^th ^to 60^th ^days of life (30-60), from 30^th ^to 90^th ^days of life (30-90), or from 60^th ^to 90^th ^days of life (60-90). Values are means ± standard error of the mean of 10 animals. Values in the same row with different superscript letters are significantly different from one another at p < 0.05 (Tukey's test)

The total caloric intake per body weight gain (kJ/100 g) was significantly lower in the H60-90, CH60-90 and CH30-90 groups compared to the C60-90 and C30-90 groups (Table 2).

The palatable hyperlipidic diet and the food cycle treatments increased the relative carcass lipid content in all of the experimental groups compared to the C groups. In addition, the H30-60 group had a higher carcass lipid content than the CH30-60 group (Table 2).

### Glucose, leptin and insulin serum concentrations

Rats exposed to the palatable hyperlipidic diet (groups H30-60, CH30-60, H30-90 and CH30-90) exhibited higher serum glucose concentrations compared to the respective chow diet-fed rats. When given the palatable hyperlipidic diet, either continuously or cycled with chow beginning on the 60^th ^day of life, the serum glucose levels were not significantly affected.

The serum leptin levels increased in all of the H groups and in the CH60-90 and CH30-90 groups compared to the respective C groups.

Both diet regimens and the different periods of treatment were effective in increasing the serum insulin concentrations in the H30-60, H60-90, CH60-90 and CH30-90 groups compared to the respective chow diet-fed rats (Table 3).

**Table 3:** Serum glucose (mg/dL), insulin (μU/mL) and leptin (ng/mL) concentrations

		C	H	CH
**Glucose**(mg/dL)	**30-60**	98.7 ± 3.6^a^	116.9 ± 3.8^b^	116.5 ± 3.6^b^
	**60-90**	95.5 ± 4.1^a^	98.3 ± 2.8^a^	104.0 ± 5.4^a^
	**30-90**	95.5 ± 4.1^a^	115.1 ± 4.4^b^	115.6 ± 4.7^b^

**Leptin**(μU/mL)	**30-60**	1.57 ± 0.19^a^	7.41 ± 1.31^b^	3.54 ± 0.32^a^
	**60-90**	3.41 ± 0.24^a^	6.67 ± 0.99^b^	6.41 ± 1.08^b^
	**30-90**	3.41 ± 0.24^a^	8.27 ± 1.01^b^	9.68 ± 1.76 ^b^

**Insulin**(ng/mL)	**30-60**	15.54 ± 1.19^a^	21.94 ± 1.81^b^	16.95 ± 1.10^a^
	**60-90**	19.10 ± 1.45^a^	25.93 ± 1.57^b^	29.81 ± 2.52^b^
	**30-90**	19.10 ± 1.45^a^	19.63 ± 2.18^a^	25.48 ± 1.09^b^

Control rats (C), rats treated with palatable hyperlipidic diet (H) and rats treated with food cycles alternating chow and palatable hyperlipidic diets (CH). The animals received these diets from 30^th ^to 60^th ^days of life (30-60), from 30^th ^to 90^th ^days of life (30-90), or from 60^th ^to 90^th ^days of life (60-90). Values are means ± standard error of the mean of 10 animals. Values in the same row with different superscript letters are significantly different from one another at p < 0.05 (Tukey's test)

### Brain glucose consumption, glucose oxidation and brain lipogenesis rate

The basal brain glucose consumption was lower in the CH30-60 group than in the C30-60 and H30-60 groups, and the insulin addition to the media normalized this parameter. Brain glucose consumption was similar between the H groups and the C groups with or without insulin addition (Table 4).

**Table 4:** Brain glucose consumption (μmol/g tissue/h) of control rats (C)

		C	H	CH
**Glucose**(μmol/g tissue/h)	**30-60**	22.52 ± 1.53^a^	20.84 ± 2.19^a^	14.59 ± 1.34^b^
	**60-90**	21.27 ± 1.47^a^	20.47 ± 1.97^a^	15.77 ± 2.10^a^
	**30-90**	21.27 ± 1.47^a^	22.46 ± 2.19^a^	19.73 ± 1.81^a^

**Glucose + Insulin**(μmol/g tissue/h)	**30-60**	21.56 ± 2.15^a^	19.87 ± 1.44^a^	18.76 ± 1.28^a^
	**60-90**	21.56 ± 1.93^a^	18.47 ± 1.43^a^	15.54 ± 1.78^a^
	**30-90**	21.56 ± 1.93^a^	20.06 ± 1.44^a^	24.35 ± 2.12^a^

Control rats (C), rats treated with palatable hyperlipidic diet (H) and rats treated with food cycles alternating chow and palatable hyperlipidic diets (CH). The animals received these diets from 30^th ^to 60^th ^days of life (30-60), from 30^th ^to 90^th ^days of life (30-90), or from 60^th ^to 90^th ^days of life (60-90). Values are means ± standard error of the mean of 10 animals. Values in the same row with different superscript letters are significantly different from one another at p < 0.05 (Tukey's test)

The brain lipogenesis rate from ^14^C-glucose, both basal levels and when stimulated by insulin, was increased in the H30-90 and CH30-90 groups compared with the C30-90 group (Table 5).

**Table 5:** Brain lipogenesis rate (^14^C-glucose incorporated in lipid/g tissue/h)

		C	H	CH
**Glucose**(^14^C-glucose incorporated in lipid/g tissue/h)	**30-60**	4.91 ± 0.22^a^	4.74 ± 0.14^a^	4.79 ± 0.14^a^
	**60-90**	4.26 ± 0.17^a^	4.96 ± 0.23^a^	4.68 ± 0.29^a^
	**30-90**	4.26 ± 0.17^a^	6.05 ± 0.20^b^	5.79 ± 0.14^b^

**Glucose + insulin**(^14^C-glucose incorporated in lipid/g tissue/h)	**30-60**	4.77 ± 0.16^a^	4.76 ± 0.17^a^	4.46 ± 0.17^a^
	**60-90**	4.53 ± 0.18^a^	4.61 ± 0.25^a^	4.63 ± 0.24^a^
	**30-90**	4.53 ± 0.18^a^	5.66 ± 0.18^b^	5.37 ± 0.10^b^

Control rats (C), rats treated with palatable hyperlipidic diet (H) and rats treated with food cycles alternating chow and palatable hyperlipidic diets (CH). The animals received these diets from 30^th ^to 60^th ^days of life (30-60), from 30^th ^to 90^th ^days of life (30-90), or from 60^th ^to 90^th ^days of life (60-90). Values are means ± standard error of the mean of 10 animals. Values in the same row with different superscript letters are significantly different from one another at p < 0.05 (Tukey's test)

The diet regimens did not alter the brain glucose oxidation in any of the experimental groups compared to the control groups (Table 6).

**Table 6:** Brain glucose oxidation (μmol/g tissue/h)

		C	H	CH
**Glucose**(μmol/g tissue/h)	**30-60**	0.66 ± 0.07^a^	0.71 ± 0.06^a^	0.71 ± 0.04^a^
	**60-90**	0.63 ± 0.07^a^	0.60 ± 0.10^a^	0.71 ± 0.07^a^
	**30-90**	0.63 ± 0.07^a^	0.70 ± 0.11^a^	0.77 ± 0.12^a^

**Glucose + Insulin**(μmol/g tissue/h)	**30-60**	0.68 ± 0.06^a^	0.56 ± 0.06^a^	0.52 ± 0.04^a^
	**60-90**	0.57 ± 0.06^a^	0.55 ± 0.11^a^	0.57 ± 0.06^a^
	**30-90**	0.57 ± 0.06^a^	0.81 ± 0.09^a^	0.61 ± 0.11^a^

Control rats (C), rats treated with palatable hyperlipidic diet (H) and rats treated with food cycles alternating chow and palatable hyperlipidic diets (CH). The animals received these diets from 30^th ^to 60^th ^days of life (30-60), from 30^th ^to 90^th ^days of life (30-90), or from 60^th ^to 90^th ^days of life (60-90). Values are means ± standard error of the mean of 10 animals. Values in the same row with different superscript letters are significantly different from one another at p < 0.05 (Tukey's test)

### Quantification of the Insulin and leptin receptors

**Figure 1:**
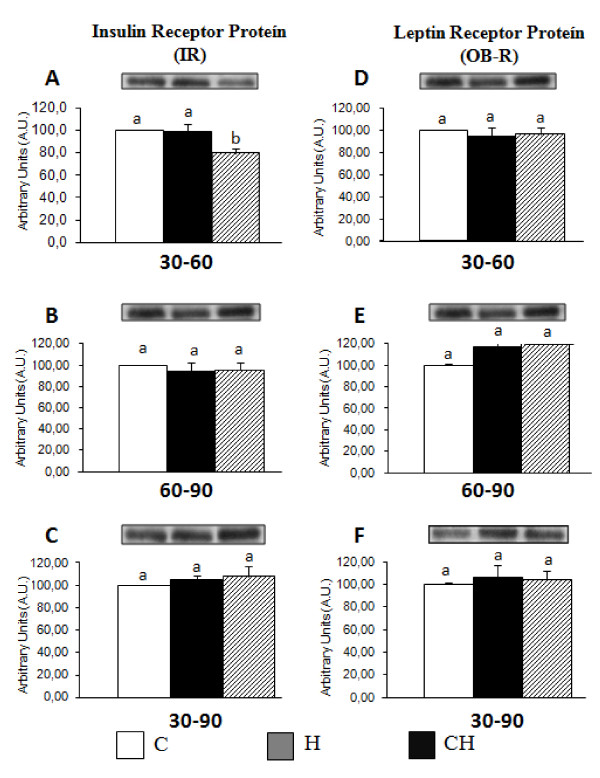
**Insulin receptor protein (IR) (A. B. and C) and leptin receptor protein (OB-R) (D. E. and F) expression (A.U. - Arbitrary Units) from hypothalamus whole tissue extract**. ***(A. D) ***groups C30-60. H30-60 and CH30-60; ***(B. E) ***groups C60-90. H60-90 and CH60-90 and ***(C. F) ***groups C30-90. H30-90 and CH30-90. Values are means ± standard error of the mean of 6 to 7 animals. Values with different superscript letters are significantly different from one another at p < 0.05 (Tukey's test).

(Figure 1A, B and 1C shows the quantification of the insulin receptor (IR) from the extracted tissue of the entire hypothalamus of the rats. The cycled diet from the 30^th ^to 60^th ^day of life significantly decreased the insulin receptor density compared to the C group.

No significant difference in leptin receptor protein levels was observed among the groups (Figure 1D, E and 1F).

## Discussion

In the present investigation, we found that both hyperlipidic diet regimes increased the carcass lipid content in all of the groups studied. We also found that when the special diet began on the 30^th ^day of life, the serum glucose level and brain glucose metabolism were more affected.

The palatable hyperlipidic diet induced a more pronounced body weight gain in the H60-90, CH60-90, and CH30-90 groups, regardless of the regimen of administration, i.e., either continuously administered or cycled with chow, although significant differences were observed based on the age and treatment period. Because caloric intake was lower in these animals, the palatable hyperlipidic diet may have increased their metabolic efficiency, as reported by others [33-35]. Similarly, successive caloric restriction and high-fat re-feeding cycles have been shown to increase metabolic efficiency and promote obesity [36].

Experimental data on the effects of palatable hyperlipidic diets are somewhat contradictory with respect to body weight gain. The collective data are not clear regarding the effects of hyperlipidic diets, also referred to as cafeteria diets, on the body weight gain of animals. Some authors have not reported an increase in body weight [19,37], whereas others have found an elevated body weight [38,39].

Both hyperlipidic diet regimes tested here increased the relative carcass lipid content in all of the studied groups. Previously, we have shown that a hyperlipidic diet administered for 8 weeks induced a more pronounced body weight gain and adiposity compared to chow diet [17]. In the present study, only 4 weeks on a hyperlipidic diet, both continuously and alternated with a chow diet, caused an enhancement in adiposity that was accompanied by an increase in serum leptin concentration, which shows that short periods of hyperlipidic diets can promote obesity. In previous studies, several obese animal models have also developed elevated circulating leptin levels [15,40].

A number of reports have demonstrated that high-fat diets may promote hyperglycemia [26,41,42]. We found higher serum glucose levels in the H30-60, CH30-60, H30-90 and CH30-90 groups than in the respective control groups. This result may suggest the occurrence of glucose intolerance. In the H60-90 and CH60-90 groups, glucose intolerance appeared to be compensated for by hyperinsulinemia, which adjusted serum glucose levels.

In our study, we did not find changes in the levels of hypothalamic leptin receptor protein, despite the observed hyperleptinemia. This finding is consistent with the results of Sahu et al. [43] and Peiser et al. [44] who also did not verify an alteration in the hypothalamus leptin receptor protein levels of rats treated with a hyperlipidic diet. In contrast, Munzberg et al. [45] studied several regions of the hypothalamus using STAT3 to map leptin-responsive cells in the brain and observed resistance to leptin action in the arcuate nucleus of the rats fed a high-fat diet.

Glucose is the major energy substrate for neurons. The major objective of our study was to analyze the effect of a hyperlipidic diet, administered either continuously or cycled with a chow diet, on brain glucose metabolism in different periods of life in the rat. There are data to suggest a that high-fat diet, especially one enriched in saturated fatty acids, decreases the learning capacity and memory of rodents and is related to insulin resistance and impaired glucose metabolism [3,4].

The administration of successive cycles of chow and high-fat diets for 30 d (CH30-60) elevated the plasma glucose levels and diminished the brain glucose consumption in relation to the C30-60 group; this was accompanied by a decrease in the levels of insulin receptor protein in the hypothalamus. A non-significant reduction (25.8%) was also observed in brain glucose consumption in the CH60-90 group compared with the C60-90 group. However, this result was not observed in the H animals or the CH30-90 group compared to the respective controls. In addition, the presence of insulin in the incubation media elevated brain glucose consumption in the CH30-90 group compared with the CH60-90 group (24.35 ± 2.12 vs. 15.54 ± 1.78, p < 0.05), which suggests that long-term treatment caused an adaptation in brain glucose metabolism.

It was reported in a review [46] that arachidonic acid, a polyunsaturated fatty acid, stimulates glucose uptake in cerebral cortical astrocytes and thus plays a role in the regulation of energy metabolism in the cerebral cortex. In the present study, the high-fat diet that was used was rich in saturated and monounsaturated fatty acids and poor in polyunsaturated fatty acids compared to the chow diet, which could promote a decrease in the incorporation of polyunsaturated fatty acids in the neuronal cell membrane and cause a decrease in glucose uptake or a defect in the expression or function of insulin receptors in the brain. Yu et al. [47] showed a positive correlation between glucose uptake by astrocytes and arachidonic acid concentration.

As described by Girard et al. [25], the concentration of GLUT1 and the rate of glucose transport in the rat brain during the suckling period are lower than the levels in adult rats. It is important to note that these changes in brain metabolism correspond to the animal's diet: milk during the suckling period, which is high in fat, or adult solid food, which is high in carbohydrates.

Duelli et al. [48] have reported a decrease in GLUT1 protein density during chronic hyperglycemia followed by an increase in brain glucose utilization. However, existing data are inconsistent with respect to the effects of hyperglycemia on brain glucose consumption. Some authors have demonstrated a decrease in brain glucose consumption [49], whereas others have shown an elevation [50,51].

It has been reported that fat transport mechanisms, oxidation and synthesis are present in some regions of the brain [52,53]. In our findings, we verified an increase in the rate of brain lipogenesis from ^14^C-glucose in the H30-90 and CH30-90 groups in media with or without insulin.

Bukato et al. [54] have shown that the activity of malic enzyme and the citrate synthase enzyme in rat brains increased gradually up to 60 days of life and was maintained at similar and stable levels until at least 160 days of life.

In summary, according to these findings, both the continuous intake of a palatable hyperlipidic diet and alternation of a high-fat intake with periods of chow intake increased the body adiposity in rats, which was independent from the duration of their administration and the age at which the altered diet was begun. In contrast, these diets caused hyperglycemia and affected brain metabolism when they were begun at an early age.

## Competing interests

The authors declare that they have no competing interests.

## Authors' contributions

DE has made substantial contributions to conception and design, carried out all the experiments, performed the statistical analysis, interpretation of data and drafted the manuscript. LMO has made substantial contributions to conception and design, analysis and interpretation of data and coordination to draft the manuscript. AAB helped to carried out the experiments and acquisition of data. CAH helped to carried out the experiments and acquisition of data. GIS carried out the fat composition of the diet and revised and helped to draft the manuscript. CSMM helped to carried out the experiments and acquisition of data. EBR participated in its design, coordination and helped to draft the manuscript. CMON has made substantial contributions to conception and design, analysis and interpretation of data, coordination and drafted the manuscript.

All authors read and approved the final manuscript.
